# Poly I:C Enhances Susceptibility to Secondary Pulmonary Infections by Gram-Positive Bacteria

**DOI:** 10.1371/journal.pone.0041879

**Published:** 2012-09-04

**Authors:** Xiaoli Tian, Feng Xu, Wing Yi Lung, Cherise Meyerson, Amir Ali Ghaffari, Genhong Cheng, Jane C. Deng

**Affiliations:** 1 Division of Pulmonary and Critical Care Medicine, David Geffen School of Medicine at UCLA, Los Angeles, California, United States of America; 2 Department of Respiratory Medicine, Second Affiliated Hospital, Zhejiang University School of Medicine, Hangzhou, Zhejiang, China; 3 Department of Microbiology, Immunology and Molecular Genetics, University of California Los Angeles, Los Angeles, California, United States of America; Louisiana State University, United States of America

## Abstract

Secondary bacterial pneumonias are a frequent complication of influenza and other respiratory viral infections, but the mechanisms underlying viral-induced susceptibility to bacterial infections are poorly understood. In particular, it is unclear whether the host's response against the viral infection, independent of the injury caused by the virus, results in impairment of antibacterial host defense. Here, we sought to determine whether the induction of an “antiviral” immune state using various viral recognition receptor ligands was sufficient to result in decreased ability to combat common bacterial pathogens of the lung. Using a mouse model, animals were administered polyinosine-polycytidylic acid (poly I:C) or Toll-like 7 ligand (imiquimod or gardiquimod) intranasally, followed by intratracheal challenge with *Streptococcus pneumoniae*. We found that animals pre-exposed to poly I:C displayed impaired bacterial clearance and increased mortality. Poly I:C-exposed animals also had decreased ability to clear methicillin-resistant *Staphylococcus aureus*. Furthermore, we showed that activation of Toll-like receptor (TLR)3 and Retinoic acid inducible gene (RIG-I)/Cardif pathways, which recognize viral nucleic acids in the form of dsRNA, both contribute to poly I:C mediated impairment of bacterial clearance. Finally, we determined that poly I:C administration resulted in significant induction of type I interferons (IFNs), whereas the elimination of type I IFN signaling improved clearance and survival following secondary bacterial pneumonia. Collectively, these results indicate that in the lung, poly I:C administration is sufficient to impair pulmonary host defense against clinically important gram-positive bacterial pathogens, which appears to be mediated by type I IFNs.

## Introduction

Viral infections of the respiratory tract are common, and frequently follow a benign clinical course. However, a significant proportion of patients develop concomitant or secondary bacterial infections [1], [2], [3], a complication that can lead to respiratory failure or death. Although children, elderly, and immunosuppressed populations are most at risk for this complication, postviral bacterial pneumonias can also occur in otherwise healthy adults resulting in substantial burden of illness. Viral-associated bacterial pneumonias have most commonly been reported following influenza infections, and have been a major cause of death during influenza pandemics of the 20^th^ century and the 2009 H1N1 influenza pandemic [4], [5], [6].

The mechanisms for how viral infections promote bacterial infections remain poorly understood, but are likely manifold and complex. In the case of influenza, mechanisms that have been proposed include influenza-induced damage of the normally protective respiratory epithelial cell layer, enhanced receptor-mediated binding of bacteria, induction of anti-inflammatory molecules such as IL-10, and attenuated expression of pattern-recognition molecules such as Toll-like receptors [7], [8], [9]. In addition, we and others have recently reported that the immune response to influenza, as represented by type I and type II IFN induction, may paradoxically suppress critical aspects of antibacterial innate immunity [10], [11], [12]. These include impairment of macrophage and neutrophil responses, two innate immune cell types that have been previously shown to be critical for clearance of bacterial pathogens from the lung. However, given that influenza infections result in a variety of alterations in the lung, whether activation of antiviral immune pathways independent of the injury induced by influenza infection can promote bacterial superinfections remains unclear.

Furthermore, it is unclear whether the phenomenon of influenza-mediated impairment of host defense is generalizable to other viral pathogens. Clinically, a number of viruses have been associated with bacterial co- or super-infections, including respiratory syncytial virus (RSV) and rhinovirus, both of which are RNA viruses [3], [13], [14] Therefore, we performed this study to test the hypothesis that simply activating viral RNA recognition receptors in the host respiratory tract would lead to impairment of bacterial clearance. We adopted an approach using synthetic compounds, specifically poly I:C, imiquimod, and gardiquimod, which are known to mimic the effects of viral nucleic acids on the immune system, followed by bacterial challenge. Poly I:C is a synthetic compound that has been shown to activate TLR3 [15], a receptor that recognizes dsRNA in the endosome, and the RIG-like receptors (RLRs) Retinoic-inducible Gene (RIG-I) and Melanoma Differentiation-associated Protein 5 (MDA5) cytoplasmic receptors that recognize RNA viral nucleic acids [16]. Imiquimod and gardiquimod (R848) activate TLR7, which recognizes single-stranded RNA [17], [18]. Both Poly I:C and TLR7 agonists are being considered as therapeutic or preventive agents for combating a variety of respiratory pathogens of pandemic or bioterrorism potential, including pandemic influenza, H5N1 avian flu, and *Francisella tularensis*, as they are believed to be an effective and safe “booster” of antiviral immune responses [19], [20], [21], [22]. In our murine model of pulmonary infection, we administered poly I:C or TLR7 agonist intranasally, followed by intratracheal challenge with common respiratory pathogens that cause postinfluenza bacterial pneumonia, to determine whether stimulation of antiviral immune pathways would increase susceptibility to secondary bacterial infection. We found that poly I:C exposure, similar to influenza infections, impairs clearance of *S. pneumoniae* and *S. aureus*. Moreover, the detrimental effects of poly I:C appear to be mediated by type I IFNs. Our findings suggest that poly I:C may not be a benign immunostimulatory molecule, and raises concern over its role as a preventive or therapeutic agent during viral pandemics.

## Methods

### Mice

Age- and sex-matched mice with a C57BL/6J genetic background were used in all experiments. *Ifnar^−/−^* mice were generated as previously reported in our lab [23]. TLR3 and Cardif (CARD-adapter Inducing IFN-β; also known as Interferon-β Promoter Simulator-1 or IPS-1, Mitrochondrial Antiviral Signaling or MAVS, and Virus Induced Signaling Adapter or VISA) double knockout mice (*Tlr3^−/−^/Cardif* ^−/−^) were generated by crossing TLR3 knockout mice (from the laboratory of Shizuo Akira, Osaka University) and Cardif knockout mice (from the laboratory of Jurg Tschopp, the University of Lausanne), both on a full C57BL/6J background, and maintained in our laboratory. Colonies of *Ifnar^−/−^*, *Tlr3^−/−^*, *Tlr3^−/−^/Cardif* ^−/−^, *Cardif* ^−/−^ and wild-type (WT) mice were maintained and housed in the same specific pathogen-free facilities at UCLA. All animal experiments were performed in accordance with NIH policies regarding the humane care and use of laboratory animals and were approved by the UCLA Office of Animal Research Oversight (OARO protocol 2005-143). Care was taken to minimize pain and suffering.

### Administration of viral TLRs ligands and IFNAR antibody

Poly I:C (#tlrl-pic), imiquimod (tlrl-imq) and gardiquimod (#tlrl-gdq) were supplied by Invivogen (San Diego, CA). Mice were anesthetized with an intraperitoneal (i.p.) injection of ketamine and xylazine mixture, and Poly I:C (50 µg), imiquimod (50 µg) or gardiquimod (100 µg) in (50 µl sterile saline or saline alone was administered intranasally. IFNAR1 neutralization was performed by administration of 1.5 mg of MAR1-5A3 (Leinco Technologies, I-401) antibody on day 1 of poly I:C, followed by booster doses of 0.75 mg on day 3 and 5 (concurrent with *S. pneumoniae* infection). Normal mouse IgG was used as control.

### Bacterial preparation and mice intratracheal (i.t.) inoculation

A serotype 3 strain of *S. pneumoniae* (ATCC 6303) was used in all experiments except where indicated otherwise. Bacteria from frozen glycerol stocks were grown in Todd-Hewitt broth at 37 degrees, 5% CO_2_ for 6 hours to mid-logarithmic phase. The culture was then spun down, and the bacterial pellet resuspended in sterile PBS. An estimated concentration was obtained by measuring the absorbance at 600 nm (A600; SmartspecTM; Bio-RAD) while the actual inoculum administered was confirmed by culturing serial 5-fold dilutions on blood agar plates overnight.

For *S. aureus* infection, a methicillin-resistant *S. aureus* strain (USA300/LAC) was kindly provided by Dr. Frank DeLeo [24]. *S. aureus* was grown overnight at 37°C in a shaking incubator (200 rpm) in tryptic soy broth (TSB). Mid-logarithmic phase bacteria were obtained after a 4 hour subculture of a 1∶200 dilution of the overnight culture. Bacterial concentrations were estimated by measuring the absorbance at 600 nm. Colony forming units (CFUs) were verified by plating dilutions of the inoculum onto TSB agar plates overnight.

Mice were anesthetized with an *i.p*. injection of ketamine and xylazine mixture. Then, the trachea was exposed, and 30 µl of the bacterial inoculum was administered via a sterile 27-gauge needle. The skin incision was closed using sutures.

### CFU determination

At the designated time points, mice were euthanized by carbon dioxide. Blood was collected in a heparinized syringe from the right ventricle, serially diluted 1∶2 with PBS, and 10 microliters plated on blood agar (for *S. pneumoniae*) or TSB plates (for *S. aureus*) to determine blood CFU. Before lung removal, the pulmonary vasculature was perfused by infusing 1 ml of PBS containing 5 mM EDTA into the right ventricle. At the designated timepoints, whole lungs were harvested and homogenized in 1 ml of PBS supplemented with protease inhibitor cocktail (Roche Applied Science). Homogenates were serially diluted 1∶5 in PBS. Ten microliters of each dilution were plated on blood agar or TSB agar plates to determine lung CFU.

### Bronchoalveolar lavage

At designated timepoints, animals were euthanized. The lungs and trachea were exposed by careful dissection. 1 ml of PBS-EDTA was instilled in the right ventricle to perfuse the pulmonary vasculature. A 23-guage polyethylene tubing was inserted into the trachea, and serial 1 ml lavages performed until a total of 10 ml had been instilled (approx. 80% return). A portion of the first ml of BAL fluid was reserved for bacterial CFU counts, and then combined with the remaining BAL fluid for cell counts. Cells in the BAL fluid were pelleted, and resuspended in RPMI for cell counting under a hemacytometer. Approximately 50,000–100,000 cells were used for cytospin preparations (Shandon) to determine cell differentials. Cytospin slides underwent staining by Diff-Quik (modified Wright-Giemsa), and number of macrophages, neutrophils, lymphocytes, and other cells were enumerated by a blinded reviewer, who counted at least 100 cells in 2 separate locations of each slide.

### Cytokine ELISA

A VerikineTM Mouse Interferon Alpha ELISA kit was purchased from PBL InterferonSource (Piscataway, NJ). Cytokine levels were measured according to the manufacturer's instructions.

### Lung histology

At designated timepoints, animals were sacrificed for collection of lung samples. 1 ml of 4% paraformaldehyde was instilled into the right ventricle to perfuse and fix the pulmonary vessels. A 23-gauge polyethylene tubing was inserted into the trachea and approximately 1 ml of 4% paraformaldehyde instilled to inflate and fix the lungs, followed by ligation of the trachea. The entire thoracic contents were excised en bloc and fixed in paraformaldehyde for 6 hours - overnight, and sent to the UCLA Translational Pathology Core Laboratory for paraffin embedding, sectioning, and H&E staining. Sections were reviewed with the assistance of a pathology faculty member, Dr. W. Dean Wallace, who was blinded to the genotype of the animals.

### Statistical analysis

Survival curves were compared using the log-rank test. For other data, statistical significance was determined using 2-tailed Mann-Whitney U test (for CFU data) or 1-way ANOVA corrected for multiple comparisons as appropriate. A *P* value of 0.05 or less was considered as statistically significant. All calculations were performed using the Prism software program for Windows (GraphPad Software Inc.). Error bars in all graphs indicate SEM and represent replicates.

## Results

### Poly I:C impairs bacterial clearance

**Figure 1:**
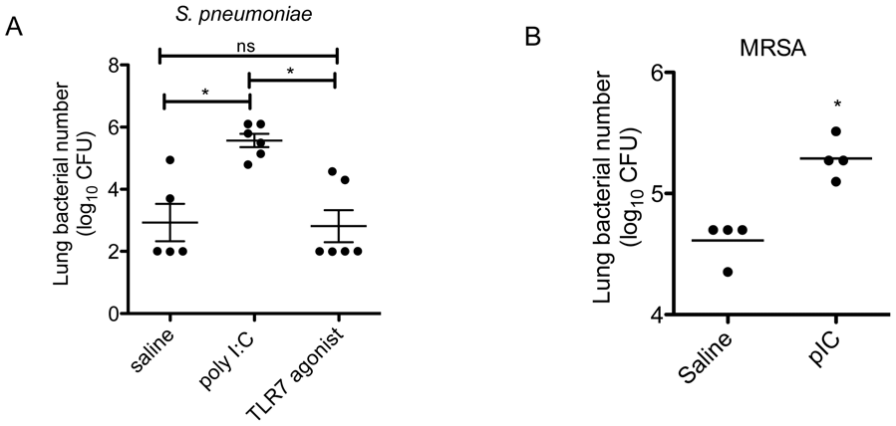
Poly I:C increases susceptibility to bacterial infection of the lung. A. Animals were administered i.n. poly I:C (50 µg), TLR7 agonist (imiquimod 50 µg or gardiquimod 50 µg) , or saline vehicle daily for 2 days. Twenty-four hours after the last dose, animals were given i.t. *S. pneumoniae* (900 CFU in 30 µl). On day 3, lungs were harvested for enumeration of CFU. *, p<0.05 compared to saline and TLR7 agonist by one-way ANOVA. Data combined from 2 separate experiments. B. Animals were administered i.n. poly I:C (50 µg) or saline vehicle daily for 3 days, followed by i.t. *S. aureus* (MRSA, LAC USA300; 6×10^6^ CFU) 24 hours later. Lungs were harvested 24 hours after bacterial infection for enumeration of CFU. *, p<0.05 compared to saline group. Data is representative of 2 independent experiments.

We first examined the effects of poly I:C administration on clearance of bacterial infections. Animals were administered intranasal poly I:C or imiquimod daily for 2 days (i.e., 2 doses). On the third day (i.e., 24 hours after the last poly I:C dose), animals were challenged with i.t. *S. pneumoniae*. We found that animals given i.n. poly I:C had significantly increased lung bacterial burdens. Interestingly, animals given imiquimod or gardiquimod (which are designated as TLR7 agonists, Figure 1A) did not demonstrate differences in bacterial clearance compared to saline-treated controls, with both groups showing robust clearance of the initial inoculum. Hence, administration of TLR7 ligands alone was not sufficient to impair bacterial clearance.

We next examined whether poly I:C also impaired clearance of another clinically important etiologic pathogen for post-viral bacterial pneumonia, methicillin-resistant *Staphylococcus aureus* (MRSA). Similarly, poly I:C pre-exposure resulted in impaired clearance of *S. aureus* at 24 hours after infection (Figure 1B). Therefore, poly I:C appears to have detrimental effects on pulmonary host defense against two clinically relevant causes of postinfluenza bacterial pneumonia, *S. pneumoniae* and MRSA.

### Duration of poly I:C pre-exposure and risk of bacterial infection

**Figure 2:**
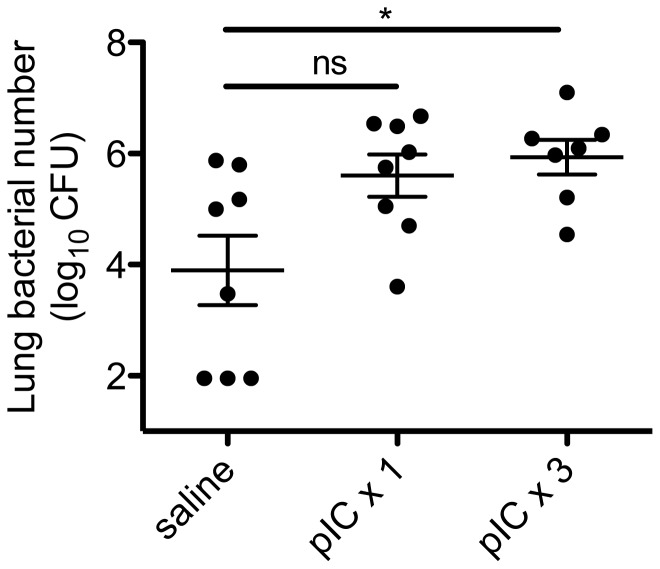
Dose-dependent effect of poly I:C on bacterial clearance from the lung. Animals were administered intranasal poly I:C (pIC, 50 µg) or saline daily for 1 or 3 days. Twenty-four hours after the last dose, animals were given i.t. *S. pneumoniae* (1×10^5^ CFU in 30 microliters). Forty-eight hours later, lungs were harvested to determine bacterial CFU. *p<0.05, poly I:C (3 doses) versus saline group. Data is combined from 2 separate experiments (n = 7–8/group).

We next examined how long animals needed to be exposed to poly I:C before developing impaired bacterial clearance. As viral infections such as influenza typically last at least several days or more, we performed studies comparing 1 dose or 3 daily doses of poly I:C to mimic the effects of ongoing viral exposure. After intranasal administration of 1 dose or 3 daily doses of poly I:C or saline vehicle, animals were challenged 24 hours later with i.t. *S. pneumoniae*. Bacterial burdens were enumerated at 48 hours. We found that there was a dose-dependent effect of poly I:C on bacterial clearance. Animals administered 1 dose of poly I:C had a trend towards impaired bacterial clearance (mean CFU 8-fold higher between poly I:C ×1 versus saline treated group, p = 0.05), whereas animals given 3 doses of poly I:C had significantly higher lung CFU (Figure 2; mean CFU 13-fold higher between poly I:C ×3 versus saline treated group, p = 0.01). Hence, the degree of impairment of bacterial clearance appeared proportional to the duration of exposure to poly I:C.

### TLR3 and Cardif pathways contribute to poly I:C-mediated host susceptibility to bacterial challenge

**Figure 3:**
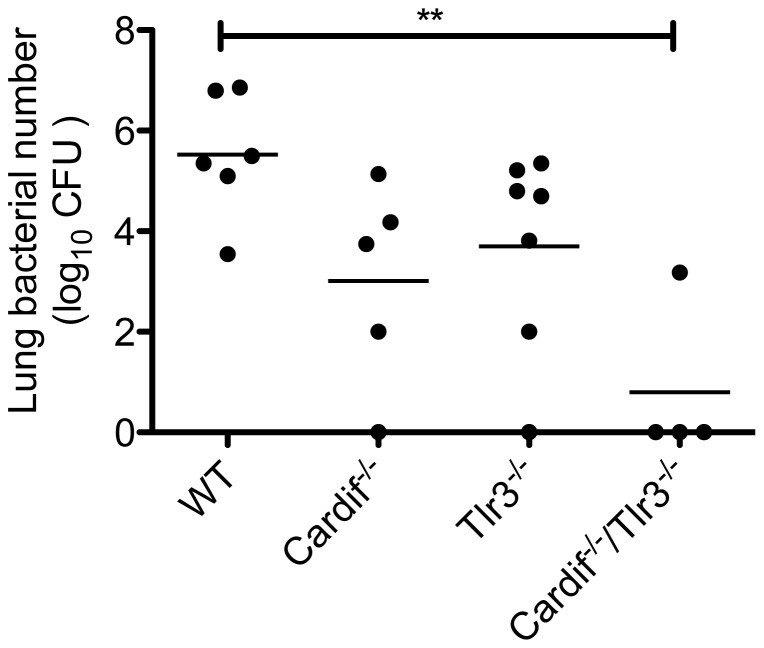
Role of viral pattern recognition pathways in mediating poly I:C-induced immunosuppression. Age- and sex-matched *Cardif* ^−/−^, *Tlr3* ^−/−^, *Cardif* ^−/−^/*Tlr3* ^−/−^ and WT animals were administered i.n. poly I:C for 3 days, followed by i.t. *S. pneumoniae* (1×10^4^ CFU). Animals were sacrificed and lungs collected at 48 hours after bacterial infection for enumeration of CFU. **, p<0.01, WT versus *Cardif* ^−/−^/*Tlr3* ^−/−^ n = 4–7/group. All other comparisons were not significant. Data is representative of 2 separate experiments.

Because poly I:C activates both TLR3 and Cardif-dependent helicases RIG-I and MDA5, we sought to determine whether either or both pathways were responsible for the detrimental effects of poly I:C on bacterial clearance. For these experiments, we administered poly I:C to animals that were deficient in either TLR3 (*Tlr3^−/−^*), Cardif (*Cardif* ^−/−^), or both (double knockouts). Cardif acts as the adaptor molecule for both RIG-I and MDA5 signaling in the initiation of antiviral responses [25], [26], [27], [28]. We found that animals deficient in either TLR3 (*Tlr3^−/−^*) or Cardif (*Cardif^−/−^*) had a trend towards improved bacterial clearance following poly I:C-pre-exposure, whereas poly I:C exposed Cardif/Tlr3 double knockout (*Cardif^−/−^*/*Tlr3^−/−^*) animals had significantly enhanced clearance of secondary bacterial infection that was comparable to saline-exposed animals. (Figure 3) In the absence of poly I:C pretreatment, however, neither *Tlr3^−/−^* nor *Cardif^−/−^* animals had significant differences in lung bacterial clearance of *S. pneumoniae* compared to wildtype animals. (Figures S1 & S2). Hence, the detrimental effects of poly I:C on bacterial clearance in our model are mediated by activation of both TLR3 and Cardif-dependent antiviral pathways.

### Effects of poly I:C exposure on late bacterial clearance

**Figure 4:**
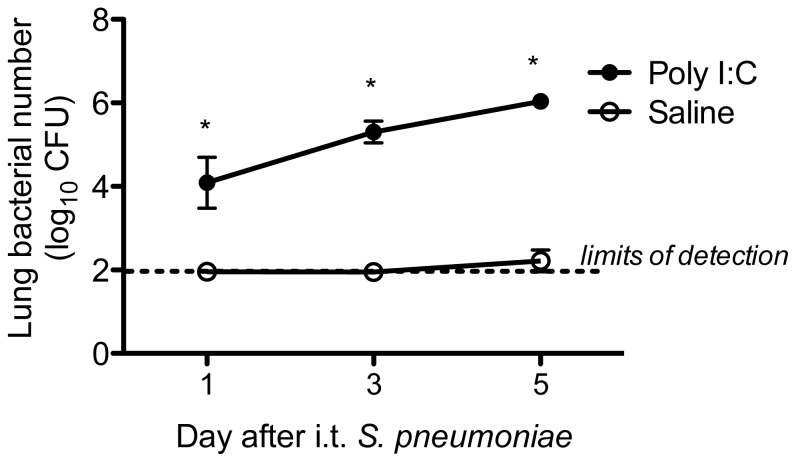
Poly I:C-pretreated animals have impaired bacterial clearance at later timepoints. Animals were administered i.n. poly I:C (50 µg) or saline daily for 3 days. Twenty-four hours after the last dose, animals were given i.t. *S. pneumoniae* (1×10^4^ CFU in 30 µl). Lungs were harvested at 1, 3, and 5 days after bacterial challenge to determine bacterial CFU, and the means of each group (n = 4/group) plotted. Error bars represent standard deviations. *p<0.05, poly I:C versus saline group at each timepoint. Data is representative of 2 separate experiments.

Given the markedly higher bacterial burdens in poly I:C-treated animals at 48 hours after pneumococcal challenge, we wished to determine whether poly I:C exposure had detrimental effects on bacterial clearance at later timepoints, or if poly I:C simply delayed kinetics of clearance. We therefore administered poly I:C for 3 daily doses, followed by i.t. *S. pneumoniae*, and assessed lung CFU at days 1, 3, and 5 after infection. We found that by day 5, saline-treated controls had lung bacterial burdens that were below our limits of detection and appeared healthy, whereas poly I:C exposed animals had increasing bacterial burdens (Figure 4) and appeared increasingly ill. Therefore, poly I:C had prolonged detrimental effects on pulmonary antibacterial defense.

### Role of type I IFNs in mediating the effects of poly I:C

**Figure 5:**
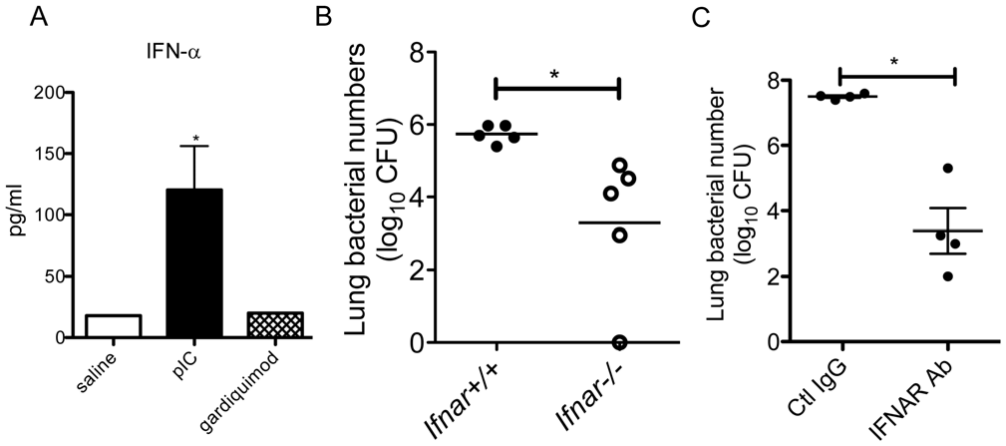
Examination of type I IFNs in poly I:C-treated animals. A. Animals were given i.n. poly I:C (pIC, 50 µg), gardiquimod (100 µg), or saline daily for 3 days. Lungs were collected for analysis of IFN-α4 in whole lung homogenates by ELISA. Mean and SD are graphed, n = 4/group. *p<0.05, poly I:C versus saline group. B. Wildtype (*Ifnar* ^+/+^) and *Ifnar* ^−/−^ animals were administered i.n. poly I:C for 3 days, followed by i.t. *S. pneumoniae* (7×10^4^ CFU). At 48 hours, lungs were collected for enumeration of CFU. n = 5/group. *p<0.05 C. Wildtype animals were administered i.n. poly I:C for 3 days (day 1, 2, and 3), followed by i.t. *S. pneumoniae* (2×10^4^ CFU) on day 4. Animals were given i.p. anti-IFNAR antibody or Control IgG (1.5 mg on day 1, 0.75 mg on day 3, and 0.75 mg on day 5). Lungs were harvested at 48 hours after *S. pneumoniae* infection. *p<0.05.

We had previously demonstrated that during influenza infection, type I IFNs elaborated during the course of viral infection led to the impaired clearance of *S. pneumoniae* [10]. We therefore sought to determine if the deleterious effects of poly I:C could also be explained by type I IFNs. We first administered i.n. poly I:C (50 µg), gardiquimod (100 µg), or saline for 3 days, and examined the levels of type I IFNs in the lung. We found that i.n. poly I:C, but not saline or gardiquimod, resulted in significant levels of IFN-α in the lung (Figure 5A). Therefore, this dose of gardiquimod was not sufficient to induce significant type I IFN levels in the lung. We next administered i.n. poly I:C for 3 doses to WT and animals deficient in type I IFN receptor (*Ifnar* ^−/−^), followed by i.t. *S. pneumoniae*, and examined bacterial clearance. We found that indeed, *Ifnar* ^−/−^ animals were protected against the detrimental effects of poly I:C, as represented by their increased ability to clear bacteria (Figure 5B). Neither group of animals had significant bacteremia at this timepoint. Because animals with congenital defects in IFNAR may acquire subtle compensatory mechanisms developmentally, we performed the same experiment using a blocking antibody for IFNAR. Following poly I:C administration, we found that the IFNAR-blocking antibody treated group had significantly improved ability to control pneumococcal challenge compared to IgG-treated controls (Figure 5C). Hence, elimination of type I IFN signaling either by genetic deletion or antibody-mediated blockade of IFNAR conferred a protective effect on bacterial clearance following poly I:C administration. We did not observe differences in bacterial counts in non-poly I:C treated WT and *Ifnar^−/−^* animals, indicating that the type I interferon pathway does not appear to mediate pulmonary clearance against *S. pneumoniae* in the absence of prior exposure to viral ligands. (Figure S1)

To examine inflammatory cell responses, we administered poly I:C for 3 daily doses, followed by i.t. administration of *S. pneumoniae* to WT and *Ifnar^−/−^* animals. At 6 hours following bacterial infection, we performed bronchoalveolar lavage to examine inflammatory cells. We found that at 6 hours, already significant differences in bacterial numbers could be observed between WT and *Ifnar^−/−^* animals. (Figure S3A) WT animals demonstrated several log-fold more bacterial CFU (mean CFU# 51,000 in WT versus 740 CFU in *Ifnar^−/−^*). Strikingly, the inflammatory response of WT animals was muted compared to the *Ifnar^−/−^* group despite having a larger bacterial load. On average, the total cell numbers (1.7×10^6^ cells in WT vs. 2.0×10^6^ in *Ifnar^−/−^*) and macrophages (1.2×10^6^ cells in WT vs. 21.3×10^6^ in *Ifnar^−/−^*) were similar between the two groups. (Figure S3B and data not shown) Although the *Ifnar^−/−^* animals had two-fold more neutrophils (4.1×10^5^ PMNs in WT vs. 8.0×10^5^ PMNs in *Ifnar^−/−^*), the differences were not significant. (Figure S3C) Hence, in this model, poly I:C-treated *Ifnar^−/−^* animals display significantly increased ability to clear pneumococcus at 6 hours following infection which is associated with a relatively modest enhancement of neutrophil recruitment compared to poly I:C treated wildtype animals.

### Poly I:C leads to increased mortality following secondary bacterial infection which is dependent on type I IFNs

**Figure 6:**
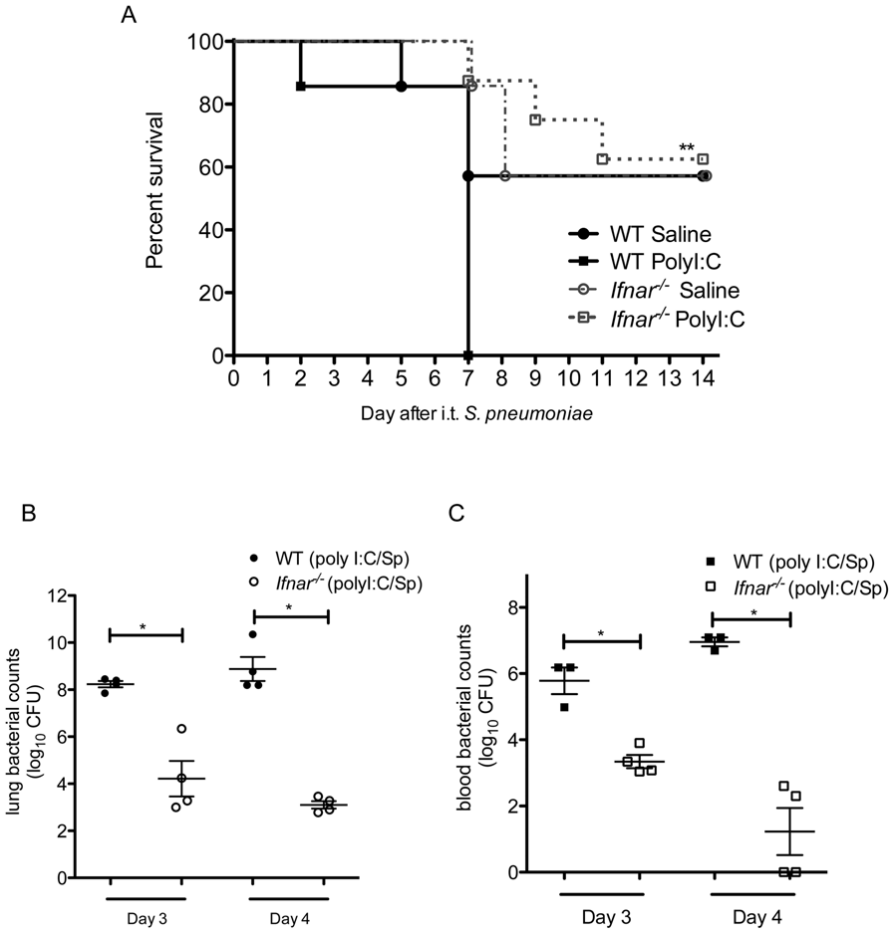
Survival and bacterial clearance at later timepoints in *Ifnar* ^−/−^ and wildtype animals following sequential poly I:C treatment and *S. pneumoniae* pulmonary infection. A. Age- and sex-matched wildtype (WT) and *Ifnar* ^−/−^ animals were administered i.n. poly I:C for 3 days, followed by i.t. *S. pneumoniae* (1×10^4^ CFU). Animals were monitored for 14 days after pneumococcal infections for indications for euthanasia due to premoribund state. **, p = 0.001 by Log-rank test, poly I:C-treated *Ifnar* ^−/−^ vs. poly I:C-treated WT group; n = 7–8/group. All other comparisons were not significant. Data is representative of 2 separate experiments. B, C. In separate experiments, age- and sex-matched wildtype (WT) and *Ifnar* ^−/−^ animals were administered i.n. poly I:C for 3 days, followed by i.t. *S. pneumoniae* (1.5×10^4^ CFU). On day 3 and 4 following bacterial infection, animals were euthanized for harvest of lungs (B) and collection of blood (C) samples, followed by enumeration of bacterial CFU. *, p<0.05; n = 4/group. Data is representative of 2 separate experiments.

Given the marked impairment in bacterial clearance by poly I:C, we wished to examine whether elimination of type I IFNs would result in improved survival following bacterial infection. We administered i.n. poly I:C or saline vehicle for 3 days, followed by i.t. *S. pneumoniae* (approximately LD_50_ dose) to WT and *Ifnar^−/−^* animals. Animals were monitored until they met criteria for euthanasia or for 14 days following *S. pneumoniae* challenge. We found that poly I:C-exposed WT animals had 100% mortality by day 7 following pneumococcal infection (Figure 6A). In contrast, poly I:C-exposed *Ifnar^−/−^* animals had 60% survival out to 14 days after bacterial challenge (p = 0.001, poly I:C treated WT versus *Ifnar^−/−^)*. No significant differences were observed between saline-treated WT and *Ifnar^−/−^* animals infected with *S. pneumoniae*. Collectively, these results demonstrate that poly I:C induces type I IFNs, which are necessary to enhance susceptibility to *S. pneumoniae* infection following activation of antiviral immunity.

In order to determine the mechanism of death, we examined lung and blood bacterial burdens at later timepoints prior to when the animals required euthanasia. We observed that WT poly I:C pretreated animals had rising bacterial CFU in the lungs and blood compared to similarly treated *Ifnar^−/−^* animals, suggesting that the absence of type I IFNs results in sustained differences in bacterial clearance following poly I:C exposure and *S. pneumoniae* infection. (Figure 6B and 6C) We then examined lung histologic sections in poly I:C treated *Ifnar^−/−^* and WT animals on day 3 following *S. pneumoniae* infection. WT animals had extensive areas of consolidation and inflammatory cell infiltrate, consistent with their enhanced bacterial loads, whereas *Ifnar^−/−^* animals had minimal areas of inflammation with mostly normal lung histology. (Figure S4) Therefore, type I IFNs appear to mediate at least in part the deleterious effects of poly I:C exposure on survival following secondary bacterial infection by impairment of early and late bacterial clearance.

## Discussion

Viral infections are a clinically significant risk factor for bacterial pneumonia. Although influenza is widely recognized to predispose hosts to secondary bacterial pneumonias, epidemiologic studies have demonstrated that other respiratory viruses also appear to be associated with bacterial pneumonias [13], [29], [30], [31], [32], [33], [34]. However, the mechanisms for this phenomenon are still poorly understood, and models testing various combinations of different viruses and bacteria are difficult to establish. Therefore, to circumvent this issue, we have adopted the approach of examining whether activation of antiviral pathways by administration of viral nucleic acid ligands common to respiratory RNA viruses (e.g., influenza, rhinovirus, RSV) could be employed as an approach to dissect out the pathways responsible for post-viral infection-mediated immune impairment. Furthermore, we wished to determine whether simply activating antiviral immune response pathways using viral RNA mimetics was sufficient to have deleterious effects on antibacterial host defense, and if so, which pathways appeared to be critical for this phenomenon.

We found that exposure to the TLR3 and RIG-I ligand, poly I:C, was sufficient to impair pulmonary clearance of secondary bacterial infection, using two clinically-relevant gram-positive pathogens, *S. pneumoniae* and MRSA, as the second “hit.” There appeared to be a dose-dependent effect of poly I:C on the level of impairment, with 1 dose of poly I:C being sufficient to observe at least a trend towards impaired clearance, but 3 daily doses of poly I:C needed to achieve statistically significant levels of impairment. Furthermore, poly I:C pre-exposure led to increased mortality rates following secondary bacterial challenge (Figure 6A), which appeared to be the result of a steadily rising lung and blood bacterial burden. (Figure 4 and 6B and 6C) The mechanism responsible for poly I:C-mediated impairment was dependent upon type I IFNs, as animals deficient in the common type I interferon receptor, *Ifnar* ^−/−^ mice, did not display the same magnitude of impaired bacterial clearance as WT following poly I:C pre-exposure. Whether type I IFN alone is sufficient to cause suppression against bacterial infection, or whether type I IFN is deleterious in the context of the immune dysregulation created by activation of “antiviral” immune responses remains to be determined. Patients treated with chronic IFN therapy often develop neutropenia and bone marrow suppression, which is the most frequent reason for dose reduction or cessation of the drug. Approximately 18–22% of subjects on interferon therapy will develop infectious complications [35], [36]. Interestingly, in these studies, infections were not necessarily associated with neutropenia, raising the possibility that chronic interferon therapy may have other detrimental effects on the immune system. In our model, we observe a relatively modest impairment of neutrophil recruitment in poly I:C treated WT versus *Ifnar^−/−^* animals; however, at this timepoint, the wildtype animals already have markedly increased bacterial numbers. Therefore, in the poly I:C model, type I IFNs may have additional effects on immune cell function beyond their effects on inflammatory cell recruitment.

In contrast, administration of comparably high or higher doses of the TLR7 agonists, imiquimod or gardiquimod, was not sufficient to suppress antibacterial host defense. We observed that the dose of gardiquimod used for intranasal challenge was not sufficient to induce significant levels of type I IFNs in the lung, supporting the notion that induction of type I IFNs is necessary for post-viral inhibition of bacterial clearance. This may be in part due to the fact that TLR7 expression is largely limited to plasmacytoid dendritic cells, whereas TLR3 expression is constitutive in the lung and more widespread, including pulmonary epithelial cells and macrophages [37]. However, if higher doses of TLR7 were used or TLR7 expression were upregulated, perhaps significant amounts of type I IFN would be elaborated which may contribute to impaired bacterial clearance. Furthermore, systemic administration of TLR7 ligands have been demonstrated to reduce the number of circulating leukocytes [38], suggesting that activation of the TLR7 pathway may have other detrimental effects on antibacterial immunity. Although in our system we failed to demonstrate a role for TLR7 activation in inducing susceptibility to bacterial pneumonia, further studies are needed to determine whether TLR7 signaling during actual influenza and other viral infections contributes towards impaired pulmonary antibacterial defense.

Viral infections have multiple effects on the host, including activation of multiple immune pathways and induction of tissue injury, which may contribute to the dysregulated immune response during secondary infections. Although poly I:C may not completely recapitulate the effects of viral infection on pulmonary host defense, we believe that our study illustrates the utility of using poly I:C as a model for viral-induced immunosuppression. Since poly I:C mimics the immune responses of viruses that either have dsRNA genomes or which undergo a dsRNA stage during replication, this model may potentially aid in the identification of molecules that would be possible candidates for “universal” mediators of postviral immunosuppression. For example, we have demonstrated now in both the influenza and the poly I:C model that type I IFNs act as a critical mediator of postviral immunosuppression against *S. pneumoniae* infection of the lung. The viral ligand approach also allows investigators to isolate the impact of specific antiviral immune pathways from the structural damage and other physical effects of a viral infection on the host organ, enhancing our understanding of how viruses promote bacterial superinfections. Based upon the results of these studies, we can next focus on the effects of TLR3, RIG-I or MDA-5 stimulation in specific cell types, which may aid in the development of targeted immunomodulatory treatments aimed at reversing the postviral immunosuppressive phenotype in critical cell populations without compromising overall antiviral immunity.

Our findings also raise concerns about using immunomodulatory therapies that boost antiviral responses as a strategy for the treatment of pandemic influenza [20]. Such an approach might protect the host from the primary viral infection only to render the host susceptible to bacterial superinfections, at least in the context of respiratory infections. Additional studies are needed to determine whether poly I:C confers increased risk of pneumonia by other types of bacteria, such as intracellular and gram-negative pathogens.

In summary, we have demonstrated that stimulation of TLR3 and Cardif-dependent pathways are sufficient to result in impaired pulmonary host defense against two clinically important gram-positive bacteria, *S. pneumoniae* and MRSA, which appears to be mediated by type I IFNs. Therefore, selective blockade of these pathways may confer protection against postviral bacterial pneumonias following influenza and other respiratory RNA viral infections.

## Supporting Information

Figure S1 **Pulmonary and blood burden following i.t.** ***S. pneumoniae*** **administration in** ***Cardif*** **^−/−^ and** ***Ifnar^−/−^*** **animals.** Age- and sex-matched *Cardif* ^−/−^, *Ifnar* ^−/−^, and WT animals were administered i.t. *S. pneumoniae* (1×10^4^ CFU). Animals were sacrificed for collection of lungs and blood at 48 hours after bacterial infection for enumeration of CFU. ns = nonsignificant for comparisons indicated; line represents lower limits of detection. Data is combined from 2 separate experiments, n = 8/group.
(TIF)

Figure S2 **Pulmonary and blood burden following i.t.** ***S. pneumoniae*** **administration in** ***Tlr3*** **^−/−^ and WT animals.** Age- and sex-matched *Tlr3* ^−/−^ and WT animals were administered i.t. *S. pneumoniae* (1×10^4^ CFU). Animals were sacrificed for collection of lungs and blood at 48 hours after bacterial infection for enumeration of CFU. ns = nonsignificant for comparisons indicated; line represents lower limits of detection. Data is combined from 2 separate experiments, n = 8–9/group.
(TIF)

Figure S3 **Early bacterial burden and inflammatory cell response in poly I:C treated animals infected with** ***S. pneumoniae*** **.** Age- and sex-matched *Ifnar* ^−/−^ and WT animals were administered i.n. poly I:C (50 µg) daily for 3 days, followed by i.t. *S. pneumoniae* (7×10^3^ CFU). At 6 hours following bacterial infection, animals underwent bronchoalveolar lavage (BAL). A. Number of bacterial CFUs were determined by serial 5-fold dilutions of the 1^st^ mL of BAL fluid. B. Total number of macrophages and neutrophils (polymorphonuclear leukocytes, or PMNs) were enumerated in total BAL cell pellets at this time point as described.
(TIF)

Figure S4 **Lung histology sections in poly I:C treated animals following** ***S. pneumoniae*** **infection.** Age- and sex-matched *Ifnar* ^−/−^ and WT animals were administered i.n. poly I:C (50 µg) daily for 3 days, followed by i.t. *S. pneumoniae* (1×10^4^ CFU). On day 3 following *S. pneumoniae* infection, lung histology sections were obtained. The WT animals showed extensive areas of consolidation, with mixed mononuclear and neutrophil infiltration. (Left panel) In contrast, *Ifnar* ^−/−^ animals had smaller, more patchy areas of inflammation, but most of the lung sections looked normal. (Right panel) Lung sections depicted are representative of each group; n = 3–4/group.
(TIF)
