# Supplementary figures and images for: Poly I:C Enhances Susceptibility to Secondary Pulmonary Infections by Gram-Positive Bacteria
Source: PLoS One. 2012 Sep 4;7(9):e41879. doi: 10.1371/journal.pone.0041879 (PMC3433467; doi:10.1371/journal.pone.0041879)

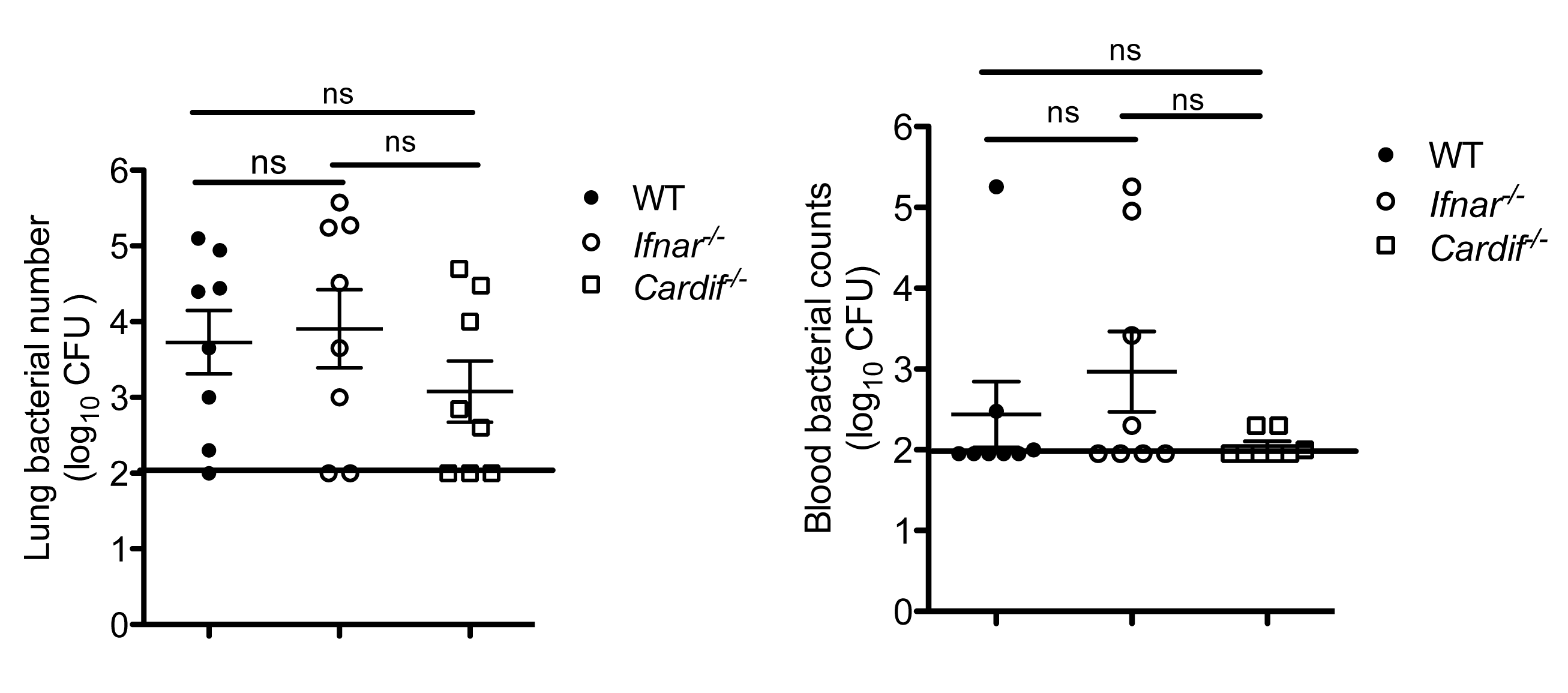

Supplement: Figure S1 — Pulmonary and blood burden following i.t. S. pneumoniae administration in Cardif −/− and Ifnar−/− animals. Age- and sex-matched Cardif −/−, Ifnar −/−, and WT animals were administered i.t. S. pneumoniae (1×10^4^ CFU). Animals were sacrificed for collection of lungs and blood at 48 hours after bacterial infection for enumeration of CFU. ns = nonsignificant for comparisons indicated; line represents lower limits of detection. Data is combined from 2 separate experiments, n = 8/group. (TIF) [file pone.0041879.s001.tif]

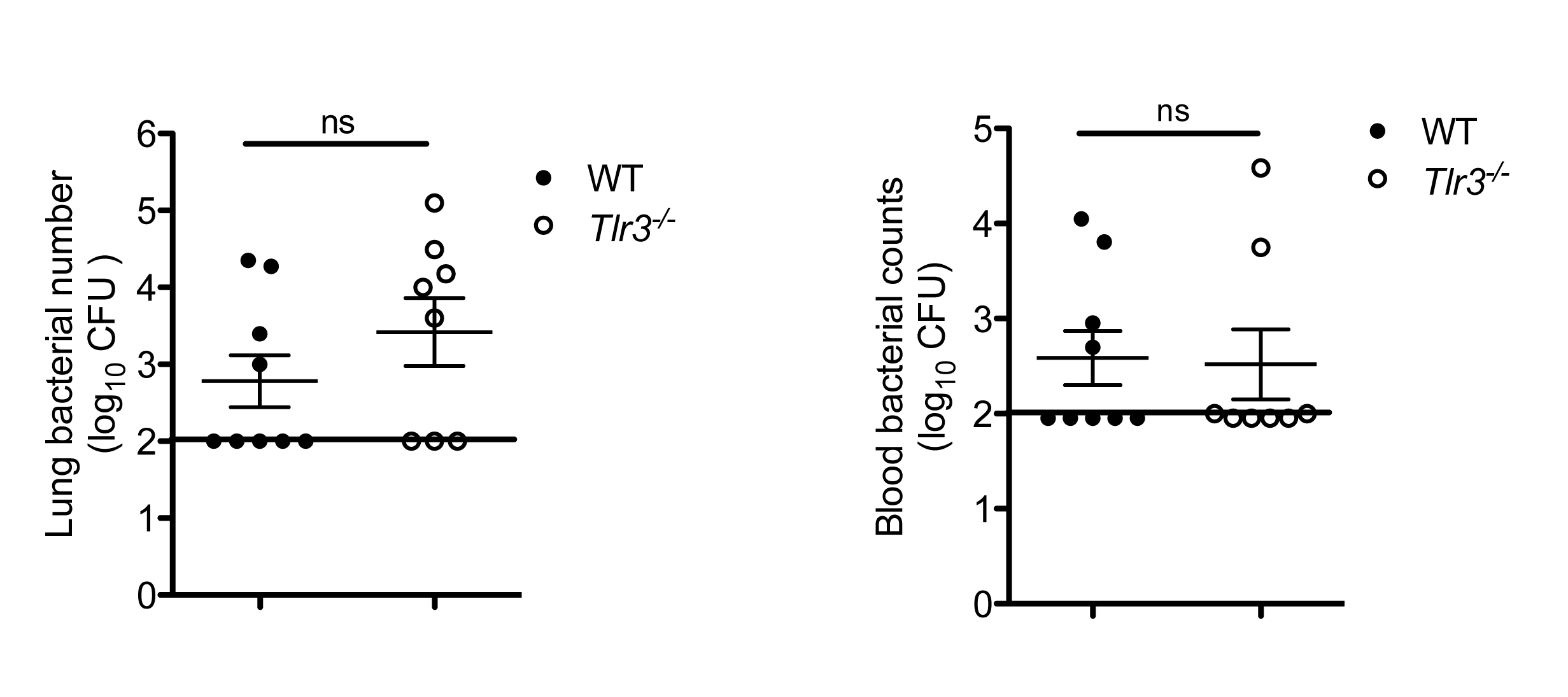

Supplement: Figure S2 — Pulmonary and blood burden following i.t. S. pneumoniae administration in Tlr3 −/− and WT animals. Age- and sex-matched Tlr3 −/− and WT animals were administered i.t. S. pneumoniae (1×10^4^ CFU). Animals were sacrificed for collection of lungs and blood at 48 hours after bacterial infection for enumeration of CFU. ns = nonsignificant for comparisons indicated; line represents lower limits of detection. Data is combined from 2 separate experiments, n = 8–9/group. (TIF) [file pone.0041879.s002.tif]

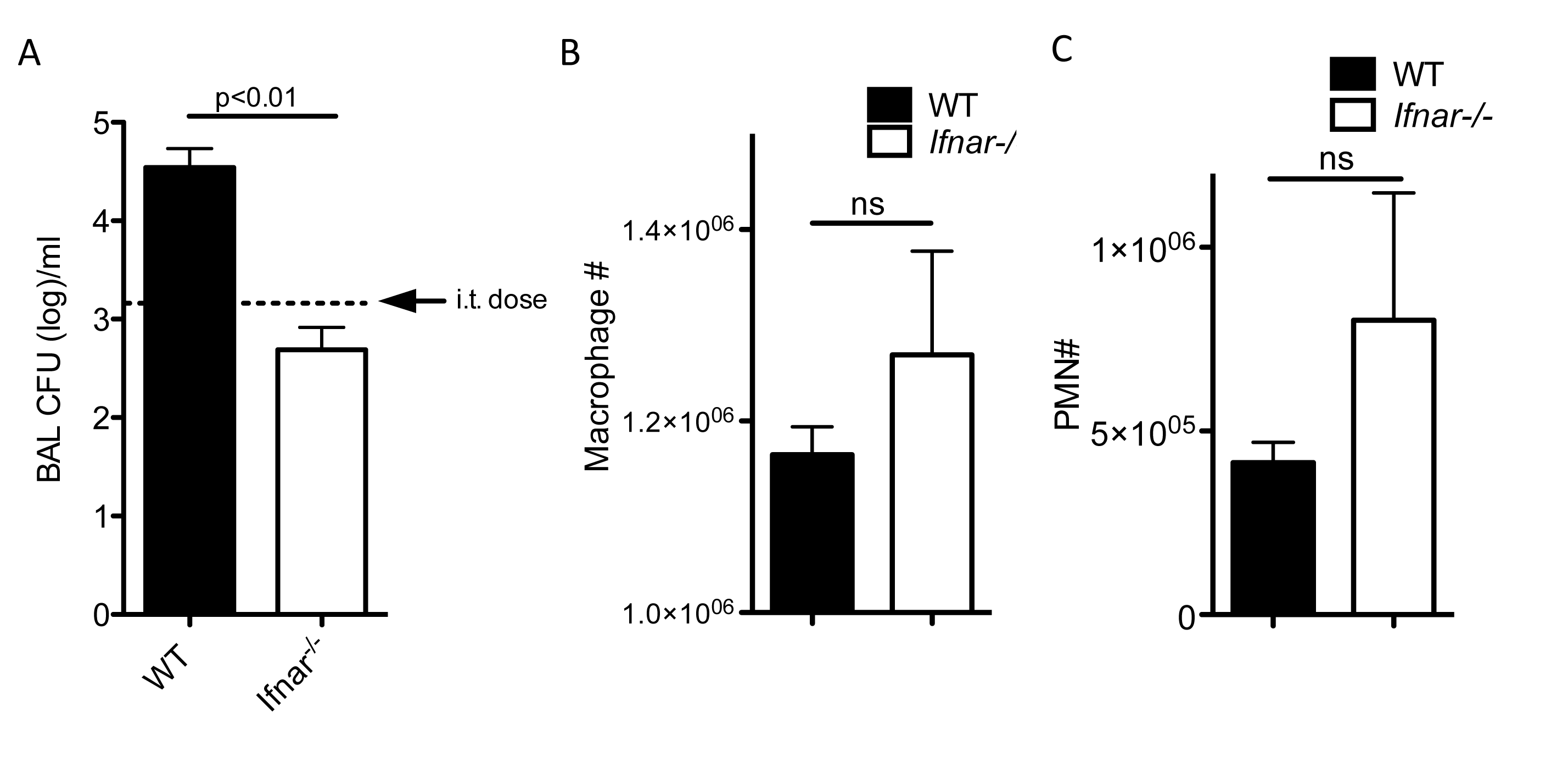

Supplement: Figure S3 — Early bacterial burden and inflammatory cell response in poly I:C treated animals infected with S. pneumoniae . Age- and sex-matched Ifnar −/− and WT animals were administered i.n. poly I:C (50 µg) daily for 3 days, followed by i.t. S. pneumoniae (7×10^3^ CFU). At 6 hours following bacterial infection, animals underwent bronchoalveolar lavage (BAL). A. Number of bacterial CFUs were determined by serial 5-fold dilutions of the 1st mL of BAL fluid. B. Total number of macrophages and neutrophils (polymorphonuclear leukocytes, or PMNs) were enumerated in total BAL cell pellets at this time point as described. (TIF) [file pone.0041879.s003.tif]

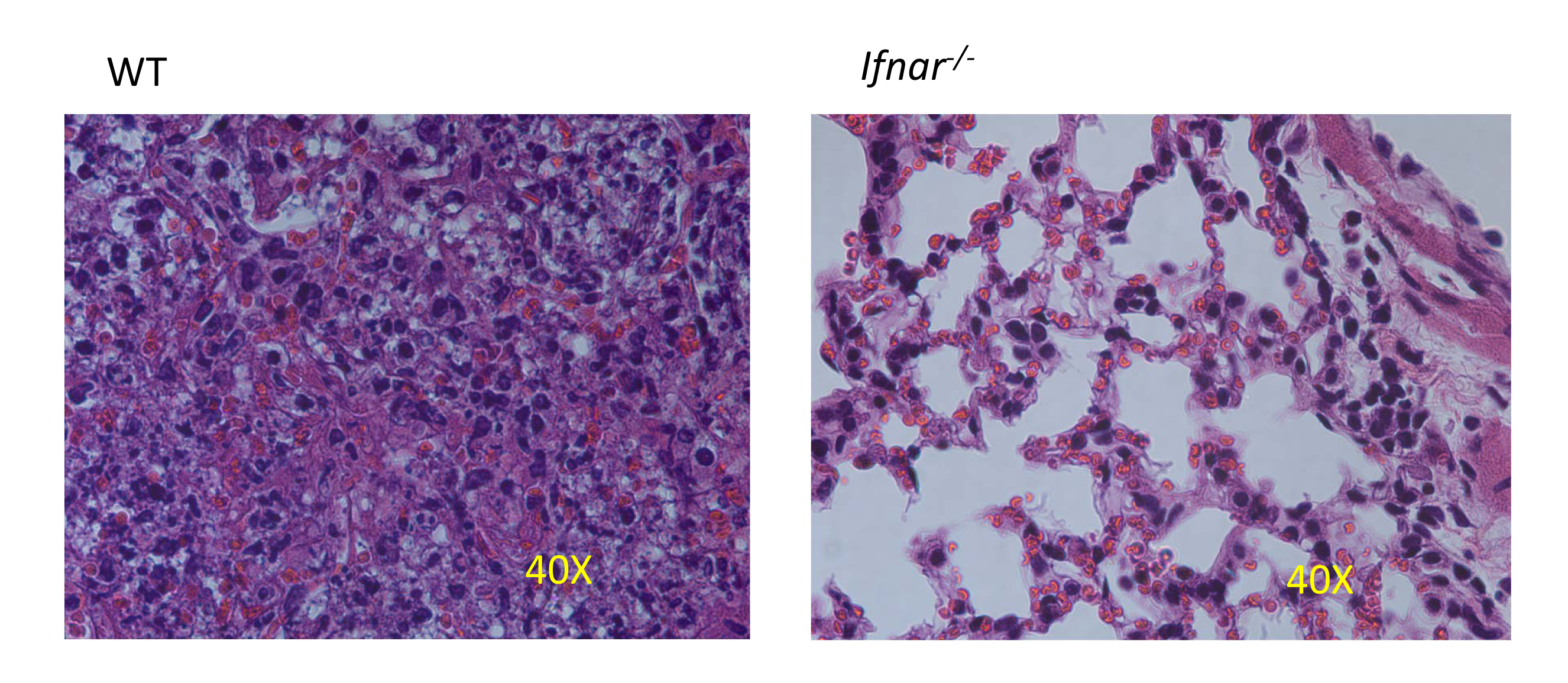

Supplement: Figure S4 — Lung histology sections in poly I:C treated animals following S. pneumoniae infection. Age- and sex-matched Ifnar −/− and WT animals were administered i.n. poly I:C (50 µg) daily for 3 days, followed by i.t. S. pneumoniae (1×10^4^ CFU). On day 3 following S. pneumoniae infection, lung histology sections were obtained. The WT animals showed extensive areas of consolidation, with mixed mononuclear and neutrophil infiltration. (Left panel) In contrast, Ifnar −/− animals had smaller, more patchy areas of inflammation, but most of the lung sections looked normal. (Right panel) Lung sections depicted are representative of each group; n = 3–4/group. (TIF) [file pone.0041879.s004.tif]
